# Igniting activation: Using unannounced standardized patients to measure patient activation in smoking cessation

**DOI:** 10.1016/j.abrep.2019.100179

**Published:** 2019-03-28

**Authors:** Jeffrey A. Wilhite, Frida Velcani, Amanda Watsula-Morley, Kathleen Hanley, Lisa Altshuler, Adina Kalet, Sondra Zabar, Colleen C. Gillespie

**Affiliations:** a Department of Medicine, Division of General Internal Medicine and Clinical Innovation, NYU School of Medicine, 462 1st Avenue, New York, NY 10016, United States of America; b Vassar College, 124 Raymond Ave, Poughkeepsie, NY 12604, United States of America; c Department of Psychiatry & Behavioral Sciences, Memorial Sloan Kettering Cancer Center, 1275 York Ave, New York, NY 10065, United States of America; d Institute for Innovation in Medical Education, Division of Quality and Evaluation and Department of Medicine, Division of General Internal Medicine and Clinical Innovation, NYU School of Medicine, 550 First Avenue, New York, NY 10016, United States of America

**Keywords:** Smoking cessation, Patient activation, Unannounced standardized patient, Medical education, Training, Counseling, Motivational interviewing, Curricula

## Abstract

**Introduction:**

Despite a decline, smoking rates have remained high, especially in communities with lower income, education, and limited insurance options. Evidence shows that physician-initiated counseling on smoking cessation is effective and saves lives, and that specific skills are needed to appropriately lead this type of patient-physician communication. Residency is a critical moment for future physicians and may be the optimal time to learn, practice, and refine this skillset. Unannounced Standardized Patients (USPs) have been found to be effective, incognito evaluators of resident practices.

**Methods:**

This study introduced rigorously trained actors (USPs) into two urban, safety-net clinics to assess resident ability to engage, activate, and counsel a pre-contemplative smoker. A complementary chart review assessed appropriate documentation in the patient's electronic health record (EHR) and its relationship to counseling style and prescribing practices.

**Results:**

Resident scores (% well done) on patient education and engagement were low (33% and 23%, respectively). Residents who coupled cessation advice with an open discussion style activated their patients more than those who solely advised cessation across all comparable measures. On EHR documentation, residents who accurately documented smoking history were more likely to directly advise their patient to quit smoking when compared to residents who did not document (t(97) = 2.828, p = .006, Cohen's D = 0.56).

**Conclusions:**

Results highlight the need to reinforce training in patient-centered approaches including motivational interviewing, counseling, and shared decision-making. Future research should focus on the effects of smokers in pre-contemplation on physician counseling style and examine the relationship between medical training and provider communication to guide interventions.

## Highlights

• Counseling, documentation, and prescribing all vary when residents meet an Unannounced Standardized Patient (USP) smoker.
• Patients are activated to quit smoking when a provider couples cessation advice with an open discussion of pros and cons.
• Training in motivational interviewing and shared decision-making can enhance patient-provider cessation communication.

## 1 Introduction

Despite declining rates of smoking in adults from 20.9% in 2005 to 15.1% in 2015, cigarette smoking remains the leading cause of preventable disease and death in the United States (Jamal et al., 2016; National Center for Chronic Disease Prevention and Health Promotion (US) Office on Smoking and Health, 2014). Rates of smoking are higher among people living below the federal poverty level, those with lower education status, and those covered by Medicaid or who are uninsured (Jamal et al., 2016; National Center for Chronic Disease Prevention and Health Promotion (US) Office on Smoking and Health, 2014). Compared with no tobacco counseling, annual counseling for adults can reduce prevalence by 3.8% (Maciosek et al., 2017). Physicians interact with at least 70% of all adult smokers in the United States every year, making them valuable sources of smoking cessation counseling and treatment (Mazor et al., 2015). Brief advice given by a doctor about quitting smoking increases the probability that a smoker will quit and maintain non-smoker status for the next 12 months, increasing the unassisted quit rate (2–3%) by an additional 1 to 3% (Lancaster, Silagy, & Fowler, 2000).

Excellent communication skills help clinicians obtain accurate information and improve patients' adherence to treatment recommendations, and this is particularly true for smoking cessation counseling (Ha & Longnecker, 2010). Studies have shown that providers using patient-centered communication can recruit smokers into treatment even if the smokers are not ready to consider the needed health behavior change (at the pre-contemplative stage of the trans-theoretical model of behavior change) (Mena, Ampadu, & Prochaska, 2017). Evidence-based recommendations suggest combining the Agency for Health Research and Quality's effective 5A approach with motivational interviewing and shared decision-making (Brown et al., 2015; Williams & Deci, 2001). The 5A steps are: *A*sk about tobacco use, *A*dvise to quit, *A*ssess willingness to quit, *A*ssist with quitting attempts, and *A*rrange for follow-up (Brown et al., 2015; Williams & Deci, 2001). Shared decision-making (SDM) and motivational interviewing aim to boost self-efficacy and autonomy while clarifying the impact that smoking has on a patient's life (Heckman, Egleston, & Hofmann, 2010; Rollnick, Butler, Kinnersley, Gregory, & Mash, 2010). These strategies share a patient-centered perspective by eliciting the patients' perspectives and internal motivation to change, rather than attempting to persuade by sharing medical information (Pocs, Hamvai, & Kelemen, 2017; Rollnick et al., 2002). Communication techniques can influence the degree of patient activation. Patient activation is the belief in the importance of being an active partner in one's own healthcare, and in having the confidence, knowledge, and skills required to manage one's own health effectively (Hibbard, Stockard, Mahoney, & Tusler, 2004). Evidence suggests that degree of patient activation in healthcare influences long-term health outcomes and that engaging and supportive communication can promote increased activation (Hibbard & Greene, 2013).

Residency training is a critical time to learn and integrate effective communication skills for lifelong practice (Charap, Levin, Pearlman, & Blaser, 2005). Evidence shows that the quality of care provided by family physicians can be traced to their postgraduate training experiences (Borgiel et al., 1989). Physicians who received training in effective communication are 3.3 times more likely to regularly counsel their patients about smoking (Merrill, Harmon, & Gagon, 2009). Furthermore, smokers are more willing to quit when counseled by residents trained in targeted communication techniques (Cornuz et al., 2002; Rollnick et al., 2002). Our smoking counseling curriculum includes a workshop on motivational interviewing, formative performance-based assessment with standardized patients, and supervision and feedback during clinical practice.

In order to evaluate our residents' approach to smoking cessation counseling, we utilized an Unannounced Standardized Patient (USP) case involving a new patient who is a smoker. USPs are actors who present as patients in actual clinical settings. They are trained to portray a standardized patient scenario in order to unobtrusively assess provider and clinic performance. This allows for data collection free from the bias associated with knowing that one is being observed (Zabar, Kachur, Kalet, & Hanley, 2013). Our medical education program has been using USPs to assess residents since 2009 (Hanley et al., 2017). By delivering a standardized case of a smoking patient into the healthcare system where residents provide ambulatory care, we sought to systematically describe: (1) how residents approach smoking cessation counseling with a new patient, including how they document the efforts; (2) whether differences in approach or treatment recommendations are associated with core general clinical skills demonstrated in the visit (including counseling, communication, and documentation); and (3) the impact of residents' smoking cessation counseling on USP ratings of patient activation.

## 2 Materials and methods

Residents had been informed during orientation to the residency program that they would be visited by USPs as part of the residency program's assessment of professionalism and clinical competence. The data collection for this study was approved by the New York University School of Medicine Institutional Review Board through a resident registry; consent is asked of residents to include their routinely-collected education data in a medical education research database. Data is reported only for those residents from whom consent was obtained (109/121 residents = 90% response rate). Study data were collected and managed using REDCap electronic data capture tools hosted at NYU Langone Medical Center (Harris et al., 2009).

### 2.1 Case and actor training

The USP is a male in his mid-40s who presented to the clinic with heartburn. He reports having smoked cigarettes since the age of 22; initially he smoked two packs per day but had cut down to one pack per day. At the time of the visit, he is in the pre-contemplative stage of quitting smoking based on the transtheoretical model of behavior change (Prochaska & Velicer, 1997). He had tried to quit cold turkey twice in the past at the request of an ex-girlfriend but returned to smoking after the relationship ended. If the resident engages him on the topic, the USP discusses his personal relationship with smoking and the possibility of quitting. See Table 1 for full details of the case.

**Table 1:** Description of USP case.

Sex, age	Male, 40–45 years old
Chief complaint	Severe and frequent heartburn
Current life situation	• Works in a restaurant/bar; lives alone in an apartment. • Did not finish high school but did get a GED. • Has never had any serious medical concerns and has always considered self in good health. • Has not had a regular check-up in over 15 years, and cannot remember the last time he had a vaccination.
Prior medical history	• No hypertension, asthma, diabetes, chest pain, shortness of breath, abdominal pain, nausea, vomiting, or diarrhea. • No surgical history.
Family medical history	• Mother has severe diabetes and is on insulin and medication. • Father had a heart attack at age 50.
Sexual history	• Has had about 10 sex partners; has not always used condoms. • Has never had an HIV test. • Was married in early twenties, but divorced after a few years • Does not have any children. • Is currently sexually active with one partner for the past 1.5 years.
Substance use	• Has been smoking since age 22, up to 2 packs per day at times. Has tried to quit twice cold-turkey at the request of an ex-girlfriend. • Currently in pre-contemplative stage of quitting smoking. • Tried marijuana in high school. • Drinks socially (3 beers on a weekend night out).
Teaching challenge for the resident	• Perform a comprehensive, evidence-based well visit. • Counsel about smoking appropriately and effectively.

The USP received 6 h of character and checklist training to ensure standardized portrayal and evaluation. Case fidelity was reviewed using discreet audio recordings from the encounter. The detection rate, which is routinely monitored by surveying residents, for this case is around 10% with the vast majority of residents only recognizing that the patient was likely to be an USP following completion of the visit. Scores do not differ by whether or not residents suspected the visit was detected.

### 2.2 Measures

Two types of assessments are used to evaluate residents in this case: 1) a comprehensive USP checklist to capture both specific practices associated with this visit and competence in core clinical skills using behaviorally-anchored items and 2) a systematic review of the visit notes written by residents in the electronic health record (EHR) (Zabar et al., 2013).

The USP checklist assesses the residents' smoking cessation counseling (5 items); core clinical competence within the domains of communication skills (12 items total across 3 sub-domains: 4 information gathering items, 5 relationship development items, and 3 patient education), patient satisfaction (4 items), and patient activation (4 items) (Hanley, Gillespie, Zabar, Adams, & Kalet, 2019). Each item of patient activation and education has descriptive behavioral anchors and is rated as not done, partly done, or well done. For analysis, these domain scores are calculated as % of items rated “well done.” A “well done” rating was indicative of a resident clearly and empathetically demonstrating the importance of smoking cessation, being clear about the impact of smoking during the encounter, or motivating a USP to quit based on their level of contemplation during the encounter. See Table 2 for details on these individual items as well as domains.

**Table 2:** Patient education, activation, and EHR documentation with internal consistency Estimates (Cronbach's alpha).^a^

USP checklist domain	Assessment items (skills)	Frequency distribution of residents for each item			Domain and total summary scores for entire sample	
		% not done (*n*)	% partially done (*n*)	% well done (*n*)	Cronbach's alpha	Mean % well done (*n* = 109)
Case specific patient education (smoking cessation counseling)	Discussed smoking risks and quitting benefits	21.1% (23)	38.5% (42)	40.4% (44)	0.747	33% SD 32%
	Explored patient's pros and cons of smoking	52.3% (57)	26.6% (29)	21.1% (23)		
	Directly advised patient to quit smoking	6.4% (7)	47.7% (52)	45.9% (50)		
	Helped patient understand importance of quitting	16.5% (18)	59.6% (65)	23.9% (26)		
Patient activation	Assessed patient's willingness to quit smoking	4.6% (5)	72.5% (79)	22.9% (25)	0.781	23% SD 31%
	Made patient want to change their smoking	30.3% (33)	55.0% (60)	14.7% (16)		
	Patient felt confident could quit smoking	45.0% (49)	36.7% (40)	18.3% (20)		
	Patient felt confident could take control of health going forward	13.8% (15)	52.3% (57)	33.9% (37)		
EHR chart review checklist domain^a^	Tobacco Use in HPI	14% (14)	–	86% (86)	N/A^a^	N/A^a^
	Tobacco Use in Problem List	43.43% (43)	–	56.57% (56)		
	Smoking Cessation Prescription	80.81% (80)	–	19.19% (19)		
	Smoking Cessation Appointment	96.97% (96)	2.02% (2)	1.01% (1)		

a Cronbach's alpha not calculated; each item assed in EHR is conceptually distinct.

Internal consistency as measured by Cronbach's alpha for each USP checklist domain are greater than 0.70. A systematic review of the residents' notes within the EHR assessed resident chart documentation (chief complaint, medical history, and quality of the note including documentation of tobacco use in the history of the present illness (HPI) field and problem list) and treatment recommendations such as appropriate smoking cessation medication and appointment prescribing practices, and these were scored as not done or done, with the exception of smoking cessation appointment being scored on a three point scale. Summary scores were not calculated for chart review domains, as each item was conceptually distinct.

## 3 Results

### 3.1 Participants

One hundred and nine internal medicine residents (61% of whom were in the Primary Care track) were visited by the USP. 35% (*n* = 38) of the residents were third year, 42% (*n* = 46) were second year, and 23% (*n* = 25) were first year. The mean length of each USP visit was 37.80 min, ranging from 15 to 95 min. Approximately half of the visits took place at an urban hospital-based clinic (*n* = 58, 53%) and half at a community-based clinic (*n* = 51, 47%). Counseling and activation scores did not significantly differ by clinic, program, or post-graduate year, and thus outcomes were aggregated across these groups.

### 3.2 Smoking cessation counseling skills

According to the USP checklist and noted in Table 2, smoking cessation counseling skills varied, with less than half (46%) of residents having directly advised patients to quit smoking and only 40% of residents having successfully discussed smoking risks and quitting benefits with their patient during the encounter.

### 3.3 Smoking cessation counseling chart documentation

**Fig. 1:**
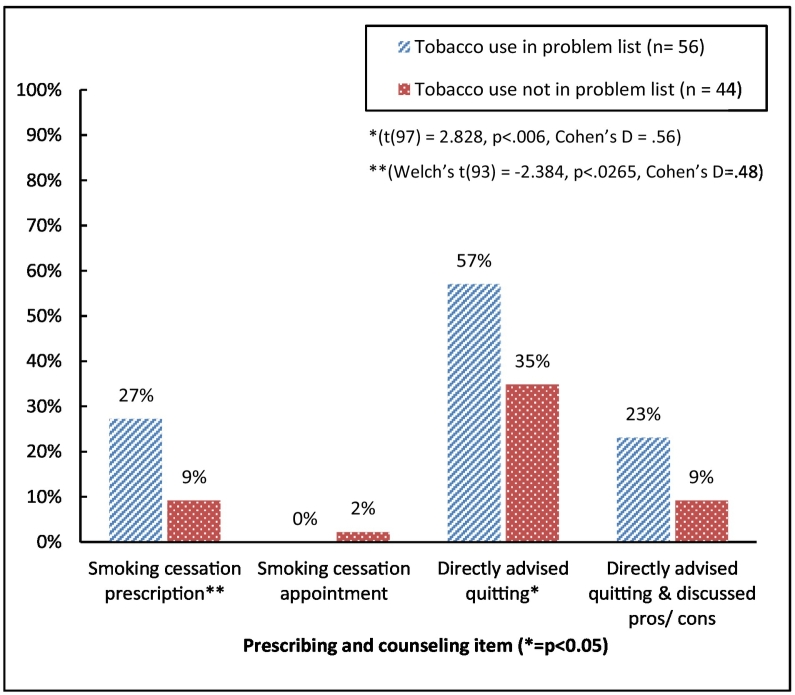
Association between documentation and prescribing & counseling style.

Of the 100 that we had EMR data on, 85% documented tobacco use (*n* = 93/109), 34% in HPI only, 6% in the problem list only, and 45% in both (*n* = 49). 7% did not document at all. As documentation in the problem list is best practice, we compared residents who documented in the problem list to those who did not. Of 60% of residents who documented tobacco use in the problem list (*n* = 56), 57% only directly advised that the USP should quit and 23% of these both directly advised quitting and discussed the pros/cons of smoking cessation. Of those who did not document tobacco use, 34% directly advised quitting while 9% both directly advised and discussed pros/cons with their patient. These results are detailed in Fig. 1.

Residents who documented use were significantly more likely to have directly advised their patient to quit than their peers who did not document in the problem list while non-documenters were more likely to prescribe tobacco replacement treatment compared to documenters (see Fig. 1).

### 3.4 Patient activation skills

As noted in Table 2, most residents failed to activate the USP to quit smoking. Less than 35% of residents scored well done on each of the four checklist items and the mean summary score for this domain was 23% well done.

### 3.5 Smoking cessation counseling and patient activation

**Fig. 2:**
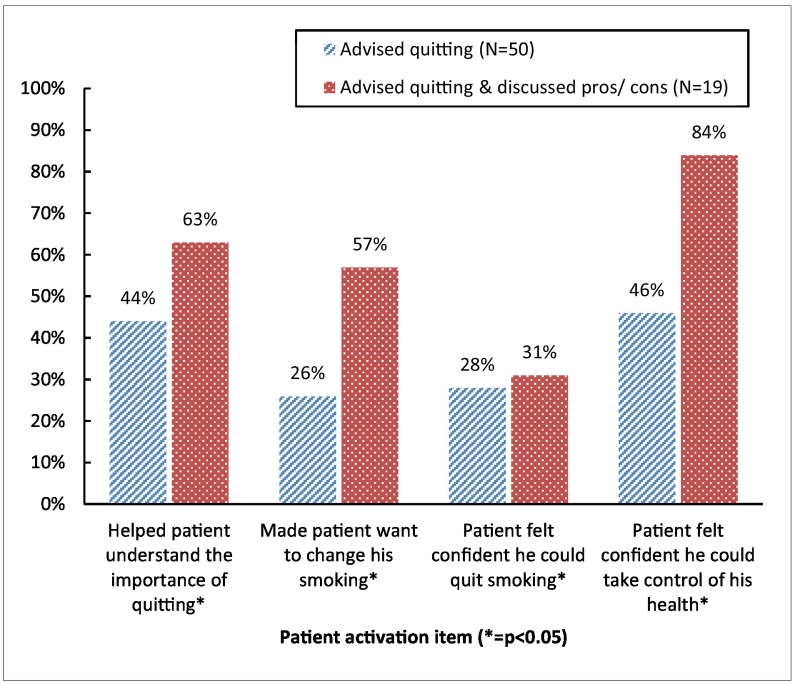
Association between residents advising quitting and patient activation.

Those residents who encouraged patients to discuss their personal relationship with smoking (pros/cons) were associated with significantly higher patient activation domain scores as well as individual items within that domain than those who did not (Fig. 2). Advising quitting and discussing pros/cons had the strongest relationship with the patient's confidence in taking control of their health. Advising quitting alone had little impact on the patient wanting to change his smoking behavior as a result of the visit.

## 4 Discussion and conclusions

This study aims to systematically describe how residents approach smoking cessation counseling and documentation, examine differences in clinical skills across residents, and understand the impact of counseling styles on patient activation. Results showed that USPs seen by residents that couple advising quitting with a discussion of pros/cons are more activated patients. Residents regularly documented tobacco use, but were routinely unlikely to prescribe or refer for nicotine cessation treatment. Low prescription rates may be indicative of resident unwillingness to counsel pre-contemplative smokers. A 2007 study found that residents are less likely to prescribe, refer, or counsel a smoker in pre-contemplation compared to other stages (Prochaska, Teherani, & Hauer, 2007). Further, while residents may have foundational knowledge of smoking, they may lack confidence or adequate cessation counseling training, making them less likely to delve into a deep discussion with a smoker (Raupach, Al-Harbi, McNeill, Bobak, & McEwen, 2014; Schkrohowsky, Kalesan, & Alberg, 2007). Research also shows that smoking cessation counseling behaviors vary by the resident's own smoker status and social circle usage of tobacco (Huang et al., 2013).

Studies have shown that the advice of a physician can be motivating and highly informative for patients previously unaware of the risks associated with smoking (Steliga, 2018); however, to counsel patients effectively physicians must have the knowledge base. Our residents may not know how to approach a pre-contemplative smoker because of a lack of personal and professional experience talking with smokers. While they likely document smoking status and advise cessation, residents may be unsure of how to take the next step and tailor their counseling based on both the patient's willingness to change their health behavior and understanding of the pros and cons of quitting.

Solely giving advice to patients is often unsuccessful at motivating behavior change (Rollnick et al., 2010). Blending motivational interviewing techniques with shared decision-making during an encounter may enhance opportunities for patient activation and treatment engagement. Previous research has shown that pre-contemplative smokers overestimate the benefits of smoking, underestimate the risks and avoid information directed to help them change, and that motivational interviewing can address these misconceptions (Rollnick et al., 2010). Enhancing motivation for patients unwilling to quit can be outlined by the “Five R's” framework: *R*elevance, *R*isks, *R*ewards, *R*oadblocks, *R*epetition, a comprehensive motivational technique for patients in early stages of readiness for change (Anczak & Nogler, 2003). When coupled with motivational interviewing, the “Five R's” enhance motivation to quit tobacco use by encouraging patients to discuss the relevance of quitting while outlining the risks of continuation, major and minor benefits of quitting, and through finally identifying barriers to quitting; routinely and repetitively (Anczak & Nogler, 2003). While physicians training sometimes incorporates motivational interviewing techniques, degree of proficiency in the technique is rarely assessed (Hall, Staiger, Simpson, Best, & Lubman, 2016).

Physicians who practice an autonomy-supportive style of counseling with opportunities for shared decision-making have been shown to increase their patients' engagement and activation during the counseling session, which in the case of smoking has been documented as having a positive relationship with increased cessation (Cornuz, 2007; Lindson-Hawley, Thompson, & Begh, 2015; Williams & Deci, 2001). In shared decision-making, patients and their clinicians cooperatively identify treatment options by navigating evidence-based medicine practices in union with preferences of the patient (Montori et al., 2011). During the discussion, patients and providers work together to make a mutually-agreed upon course of action, providing new opportunity for the development of patient autonomy (Montori et al., 2011). The power of autonomy-focused counseling style was supported by our finding of increased patient activation in USPs after seeing providers who discussed both the pros and cons of smoking.

Promoting patient activation enhances the clinician-patient experience and is correlated with better health outcomes (Greene & Hibbard, 2012). In the current study, the coupling of advising and discussing had the greatest positive impact on the patients' confidence in controlling their own health. Patients who report that their provider helped them in concrete and specific ways were more activated than patients who reported the contrary (Glasgow et al., 2005; Parchman, Zeber, & Palmer, 2010). Providers can activate patients by helping them learn to monitor their health, set goals, and/or set up cessation methods (Glasgow et al., 2005; Parchman et al., 2010). Activated patients have increased chances of receiving preventive care, and have lower rates of smoking and lower BMI (Greene & Hibbard, 2012). Including an assessment of mastery of motivational interviewing and shared decision-making training during medical education may be crucial to enriching patient-provider communication, and in turn, enhancing the opportunity for patient engagement and activation in smoking cessation treatment. Further, introducing counseling approaches that can be tailored to correspond with a patient's stage of readiness for behavior change may fill gaps in care that arise due to a patient's limited motivation to change. Motivating patients to take control of their health while also providing the opportunity for their engagement in treatment may be the key to lasting smoking cessation.

There are specific challenges associated with the use of USPs in evaluating clinicians. Residents may modify their practice behaviors if they suspect the identity of a USP (Siminoff et al., 2011). Provider engagement in discussion and documentation preferences may impede proper documentation of a patient's smoking-cessation needs in the EHR. Additionally, USPs are only able to provide a snapshot of a one-time assessment of clinical skills. Other environmental factors including time constraints faced by provider, chaos of clinic, and EHR skills may impact outcomes. Further, we did not assess resident smoker status and their social circle tobacco usage. This data may not be generalizable to the entire clinic population, as the study is an in-depth assessment of one standardized patient's experience with a range of clinicians in an urban, safety-net clinic system.

Given that evidence shows that residents continue their clinical practice through attending practice, new ways to engage residents in changing their smoking cessation communication skills and behaviors should be explored. The current use of in-the-moment feedback during clinical supervision, lectures, and some motivational interviewing practice and assessment is not eliciting behavior change. Residents should receive regular audits and feedback on their smoking cessation practice coupled with feedback from their patients on needed improvement areas. Residents could also benefit from enhanced, standardized training in tailoring communication to a patient's stage of readiness, and in understanding the importance of including discussion of pros and cons during counseling regardless of a patient's stage or a provider's smoker status. In addition to clinical system-level changes, easier referral and support staff helping to initiate discussions of pros and cons to smoking combined with residents' increased awareness of effective smoking cessation skills and materials may be necessary to improve the health of our patients.

## Conflict of interest

All authors declare that they have no conflicts of interest to disclose.

## Role of Funding Sources

This work was supported by the Agency for Healthcare Research and Quality (AHRQ 5 R18 HS 021176–02) and the Health Resources & Services Administration (HRSA 15-A0-00-004497).

## References

[bb0005] Anczak J.D., Nogler R.A. (2003). Tobacco cessation in primary care: Maximizing intervention strategies. Clinical Medicine & Research.

[bb0010] Borgiel A.E., Williams J.I., Bass M.J., Dunn E.V., Evensen M.K., Lamont C.T., Spasoff R.A. (1989). Quality of care in family practice: Does residency training make a difference?. Canadian Medical Association Journal.

[bb0015] Brown C.H., Medoff D., Dickerson F.B., Fang L.J., Lucksted A., Goldberg R.W., Dixon L.B. (2015). Factors influencing implementation of smoking cessation treatment within community mental health centers. Journal of Dual Diagnosis.

[bb0020] Charap M.H., Levin R.I., Pearlman R.E., Blaser M.J. (2005). Internal medicine residency training in the 21st century: Aligning requirements with professional needs. The American Journal of Medicine.

[bb0025] Cornuz J. (2007). Smoking cessation interventions in clinical practice. European Journal of Vascular and Endovascular Surgery.

[bb0030] Cornuz J., Humair J., Seematter L. (2002). Efficacy of resident training in smoking cessation: A randomized, controlled trial of a program based on application of behavioral theory and practice with standardized patients. Annals of Internal Medicine.

[bb0035] Glasgow R.E., Wagner E.H., Schaefer J., Mahoney L.D., Reid R.J., Greene S.M. (2005). Development and validation of the patient assessment of chronic illness care (PACIC). Medical Care.

[bb0040] Greene J., Hibbard J.H. (2012). Why does patient activation matter? An examination of the relationships between patient activation and health-related outcomes. Journal of General Internal Medicine.

[bb0045] Ha J.F., Longnecker N. (2010). Doctor-patient communication: A review. Ochsner Journal.

[bb0050] Hall K., Staiger P.K., Simpson A., Best D., Lubman D. (2016). After 30 years of dissemination, have we achieved sustained practice change in motivational interviewing?. Addiction Journal.

[bb0055] Hanley K., Gillespie C., Zabar S., Adams J., Kalet A. (2019). Monitoring communication skills progress of medical students: Establishing a baseline has value, predicting the future is difficult. Patient Education and Counseling.

[bb0060] Hanley K., Zabar S., Altshuler L., Lee H., Ross J., Rivera N., Gillespie C. (2017). Opioid vs nonopioid prescribers: Variations in care for a standardized acute back pain case. Substance Abuse.

[bb0065] Harris P.A., Taylor R., Thielke R., Payne J., Gonzalez N., Conde J.G. (2009). Research electronic data capture (REDCap)–a metadata-driven methodology and workflow process for providing translational research informatics support. Journal of Biomedical Informatics.

[bb0070] Heckman C.J., Egleston B.L., Hofmann M.T. (2010). Efficacy of motivational interviewing for smoking cessation: A systematic review and meta-analysis. Tobacco Control.

[bb0075] Hibbard J.H., Greene J. (2013). What the evidence shows about patient activation: Better health outcomes and care experiences; fewer data on costs. Health Affairs.

[bb0080] Hibbard J.H., Stockard J., Mahoney E.R., Tusler M. (2004). Development of the patient activation measure (PAM): Conceptualizing and measuring activation in patients and consumers. Health Services Research.

[bb0085] Huang C., Guo C., Yu S., Feng Y., Song J., Eriksen M., Koplan J. (2013). Smoking behaviours and cessation services among male physicians in China: Evidence from a structural equation model. Tobacco Control.

[bb0090] Jamal A., King B.A., Neff L.J., Whitmill J., Babb S.D., Graffunder C.M. (2016). Current cigarette smoking among adults - United States, 2005–2015. MMWR. Morbidity and Mortality Weekly Report.

[bb0095] Lancaster T., Silagy C., Fowler G. (2000). Training health professionals in smoking cessation. Cochrane Database of Systematic Reviews.

[bb0100] Lindson-Hawley N., Thompson T.P., Begh R. (2015). Motivational interviewing for smoking cessation. Cochrane Database of Systematic Reviews.

[bb0105] Maciosek M.V., LaFrance A.B., Dehmer S.P., McGree D.A., Xu Z., Flottemesch T.J., Solberg L.I. (2017). Health benefits and cost-effectiveness of brief clinician tobacco counseling for youth and adults. Annals of Family Medicine.

[bb0110] Mazor K.M., Jolicoeur D., Hayes R.B., Geller A.C., Churchill L., Ockene J.K. (2015). Assessing medical students' tobacco dependence treatment skills using a detailed behavioral checklist. Teaching and Learning in Medicine.

[bb0115] Mena J.A., Ampadu G.G., Prochaska J.O. (2017). The influence of engagement and satisfaction on smoking cessation interventions: A qualitative study. Substance Use & Misuse.

[bb0120] Merrill R., Harmon T., Gagon H. (2009). Physician-based tobacco smoking cessation counseling in Belgrade, Serbia. International Electronic Journal of Health Education.

[bb0125] Montori V.M., Shah N.D., Pencille L.J., Branda M.E., Van Houten H.K., Swiglo B.A., Wermers R.A. (2011). Use of a decision aid to improve treatment decisions in osteoporosis: The osteoporosis choice randomized trial. The American Journal of Medicine.

[bb0130] National Center for Chronic Disease Prevention and Health Promotion (US) Office on Smoking and Health (2014). The health consequences of smoking-50 years of progress: A report of the surgeon general.

[bb0135] Parchman M.L., Zeber J.E., Palmer R.F. (2010). Participatory decision making, patient activation, medication adherence, and intermediate clinical outcomes in type 2 diabetes: A STARNet study. Annals of Family Medicine.

[bb0140] Pocs D., Hamvai C., Kelemen O. (2017). Health behavior change: Motivational interviewing. Orvosi Hetilap.

[bb0145] Prochaska J., Teherani A., Hauer K.E. (2007). Medical students' use of the stages of change model in tobacco cessation counseling. Journal of General Internal Medicine.

[bb0150] Prochaska J., Velicer W.F. (1997). The transtheoretical model of health behavior change. American Journal of Health Promotion.

[bb0155] Raupach T., Al-Harbi G., McNeill A., Bobak A., McEwen A. (2014). Smoking cessation education and training in UK medical schools: A national survey. Nicotine & Tobacco Research.

[bb0160] Rollnick S., Butler C.C., Kinnersley P., Gregory J., Mash B. (2010). Motivational interviewing. British Medical Journal.

[bb0165] Rollnick S., Seale C., Kinnersley P., Rees M., Butler C., Hood K. (2002). Developing a new line of patter: Can doctors change their consultations for sore throat?. Medical Education.

[bb0170] Schkrohowsky J.G., Kalesan B., Alberg A.J. (2007). Tobacco awareness in three U.S. medical schools. Journal of Addictive Diseases.

[bb0175] Siminoff L.A., Rogers H.L., Waller A.C., Harris-Haywood S., Esptein R.M., Carrio F.B., Longo D.R. (2011). The advantages and challenges of unannounced standardized patient methodology to assess healthcare communication. Patient Education and Counseling.

[bb0180] Steliga M.A. (2018). Smoking cessation in clinical practice: How to get patients to stop. Seminars in Thoracic and Cardiovascular Surgery.

[bb0185] Williams G.C., Deci E.L. (2001). Activating patients for smoking cessation through physician autonomy support. Medical Care.

[bb0190] Zabar S., Kachur E., Kalet A., Hanley K. (2013). Objective structured clinical examinations.

